# A simple method to reduce infection of ventriculoperitoneal shunts

**DOI:** 10.1186/1743-8454-7-S1-S45

**Published:** 2010-12-15

**Authors:** Tausif Rehman, Atiq-ur Rehman, Hassaan Bashir, Vikas Gupta

**Affiliations:** 1 Department of Neurosurgery University of New Mexico 2211 Lomas Boulevard Albuquerque, NM 87131, USA; 2 Department of Neurosurgery PO Box 2290, King Fahd Specialists Hospital Al-Qassim, Saudi Arabia; 3 Department of Medicine, Aga Khan University P.O.Box 3500, Stadium Road Karachi, 74800, Pakistan; 4 Department 0f Neurology University of New Mexico 2211 Lomas Boulevard Albuquerque, NM 87131, USA

## Background

Post-operative shunt infection is the most common and feared complication of ventriculoperitoneal shunt placement for treatment of hydrocephalus. The rate of shunt infection is highest in the first postoperative month. The most common organisms responsible for shunt infection include coagulase-negative staphylococcus and staphylococcus aureus. This suggests a transfer of patient’s skin flora via the surgeons’ glove as a possible means of infection. This led to our hypothesis of changing gloves before handling the shunt catheter as a simple way to reduce post-operative shunt infections.

## Materials and methods

A total of 111 neonates born with congenital hydrocephalus requiring VP shunt placement were prospectively enrolled and divided into two groups. Group A, the control, had 54 neonates treated with standard protocol VPS placement while Group B had 57 neonates in which gloves were changed before the shunt catheter was handled. Shunt infection rates were compared up to six months postoperatively.

## Results

There was a statistically significant reduction of infection rate from 16.33% in Group A; the control, to 3.77% in Group B.

## Conclusions

Our study shows that a change of gloves before handling the shunt catheter may be a simple and cost effective way to reduce the burden of postoperative shunt infections. Our study was limited by a small sample size. A larger study is required to evaluate the effectiveness of this simple measure to reduce post operative shunt infections.

